# Morphological and Phylogenetic Analysis of a New Jellyfish of *Phyllorhiza* (Scyphozoa, Mastigiidae) from the East China Sea

**DOI:** 10.3390/biology14060632

**Published:** 2025-05-29

**Authors:** Xiaoyin Chen, Zhijie Hu, Zifeng Zhan, Yaojun Chen, Sirong Mu, Bingpeng Xing, Kuidong Xu

**Affiliations:** 1 Laboratory of Marine Organism Taxonomy and Phylogeny, Qingdao Key Laboratory of Marine Biodiversity and Conservation, Shandong Province Key Laboratory of Experimental Marine Biology, Institute of Oceanology, Chinese Academy of Sciences, Qingdao 266071, China; chenxiaoyin@tio.org.cn (X.C.); zzhan@qdio.ac.cn (Z.Z.); 2 Third Institute of Oceanography, Ministry of Nature Resources, Xiamen 361102, China; 3 University of Chinese Academy of Sciences, Beijing 100049, China; 4 Ningbo Ocean World, Ningbo 315000, China; 2012091154@nbu.edu.cn (Z.H.);; 5 Taizhou Bureau of Natural Resources and Planning, Taizhou 318000, China

**Keywords:** jellyfish biodiversity, life history, *Phyllorhiza*, mitochondrial gene, morphological and molecular taxonomy, East China Sea

## Abstract

A new species of jellyfish, *Phyllorhiza yurena* Chen, Hu & Xing sp. nov., is described from the East China Sea based on an integrative approach combining morphological and molecular data. Specimens were collected from coastal waters and cultured in the laboratory to observe their complete life cycle, including polyps, ephyrae, and medusae. Distinct morphological features such as the blue lappet stems in ephyrae, white warts on the exumbrella, and multiple short club-shaped appendages on the mouth arms distinguish the new species from its congeners. Phylogenetic analyses based on mitochondrial (*COI*, *16S*) and nuclear (*18S*, *28S*) gene markers confirmed its taxonomic distinctiveness, with *COI* and *16S* markers providing the most resolution. The study highlights the importance of combining life history observations with molecular tools for accurate jellyfish taxonomy and provides a revised identification key for the genus *Phyllorhiza*. This discovery contributes to the understanding of jellyfish biodiversity in the East China Sea and offers a baseline for future ecological and biogeographic studies.

## Simple Summary

Jellyfish of the genus *Phyllorhiza* are ecologically significant marine organisms that have recently been observed in increasing numbers in the East China Sea. These jellyfish can have substantial impacts on marine ecosystems, including influencing food webs and affecting aquaculture. In this study, we describe a new species of *Phyllorhiza*, named *Phyllorhiza yurena* Chen, Hu & Xing sp. nov., based on detailed morphological and molecular analyses. We highlight the importance of understanding the life cycle stages of jellyfish to accurately identify and classify species, which is crucial for ecological research and resource management.

## 1. Introduction

Jellyfish, a key group of zooplankton, serve pivotal roles in marine ecosystems. They act as both prey for higher trophic levels (e.g., fish and turtles) and as voracious predators, directly shaping food web dynamics and nutrient cycling processes [1,2]. However, population outbreaks of jellyfish increasingly threaten coastal human activities. For instance, jellyfish aggregations can clog fishing nets, reduce catch efficiency, and damage equipment in commercial fisheries. In aquaculture systems, jellyfish stinging cells (nematocysts) inflict physical injuries on farmed fish, increasing susceptibility to infections and mortality. Notably, detached tentacles remain biologically active post-outbreak, posing prolonged risks to aquaculture operations [3]. Beyond fisheries, jellyfish blooms disrupt tourism by forcing beach closures and impair coastal power plants through blockage of cooling water intakes, occasionally triggering operational shutdowns. Of particular concern are invasive jellyfish species such as *Phyllorhiza punctata* von Lendenfeld, 1884, which is originally native to the Pacific Ocean; this species has colonized the Gulf of Mexico, where its rapid proliferation disrupts local ecosystems and imposes significant economic costs via predation on fish larvae and competition with native species.

Jellyfish exhibit complex and variable life cycles, with most species undergoing a life cycle that alternates between sexual and asexual reproduction stages [4,5]. During the life cycle, polyps reproduce asexually by budding and through the process of strobilation, in which ephyrae are produced by transverse fission. Jellyfish taxonomy has historically been fraught with challenges due to their complex life cycles, morphological plasticity, and the difficulty of observing key features in the field. Integrated taxonomy provides multiple lines of evidence to resolve these uncertainties [6]. For instance, the freshwater jellyfish *Craspedacusta sowerbii* Lankester, 1880 has been widely studied, but its global distribution and taxonomic status remain unclear due to the ephemeral medusa stage and the lack of comprehensive genetic studies. Lüskow et al. accurately defined species boundaries and understood their evolutionary history by combining morphological observations with molecular analyses [7].

The genus *Phyllorhiza* Agassiz, 1862 comprises a group of Rhizostome jellyfish distinguished by their characteristic bluish pigmentation and symbiotic association with zooxanthellae [8]. Despite their ecological importance in coastal and estuarine ecosystems, species within this genus display remarkable morphological plasticity across different life cycle stages, presenting considerable challenges for accurate taxonomic identification [9,10,11,12]. This complexity is further compounded by the limited availability of comprehensive life cycle studies that integrate molecular data with morphological development, thereby impeding our understanding of their biogeographic patterns.

In recent years, a commonly encountered *Phyllorhiza* species has been frequently observed in the East China Sea, with a notable bloom occurring in June 2023. In this study, we conducted a systematic investigation of this species, examining its ontogenetic morphological transitions—from polyps to medusae—and analyzing its phylogenetic placement based on DNA markers. Through the integration of morphological and molecular evidence, we describe and illustrate a new species, *Phyllorhiza yurena* Chen, Hu & Xing, sp. nov., and provide an identification key for all recognized species within the genus *Phyllorhiza*.

## 2. Materials and Methods

### 2.1. Sample Collection and Morphological Examination

**Figure 1:**
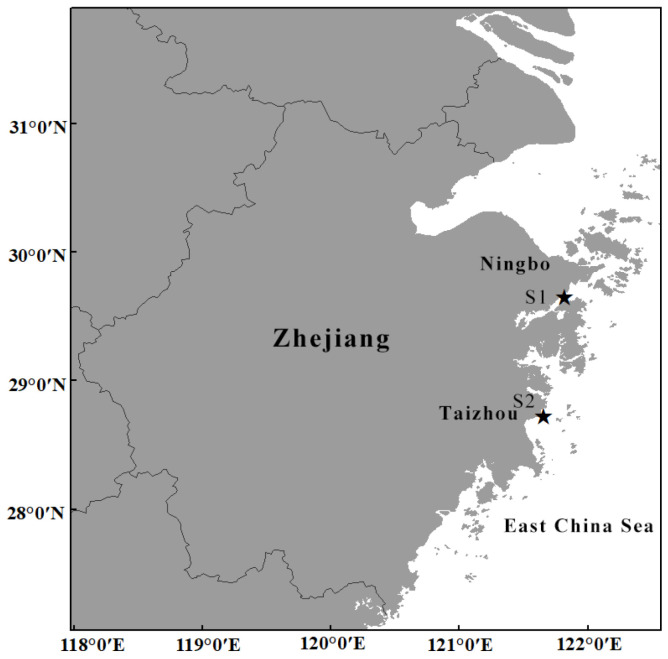
Sampling locations of *Phyllorhiza yurena* Chen, Hu & Xing sp. nov. S1. Sampling location of polyps; S2. Sampling location of adult medusae paratype.

**Figure 2:**
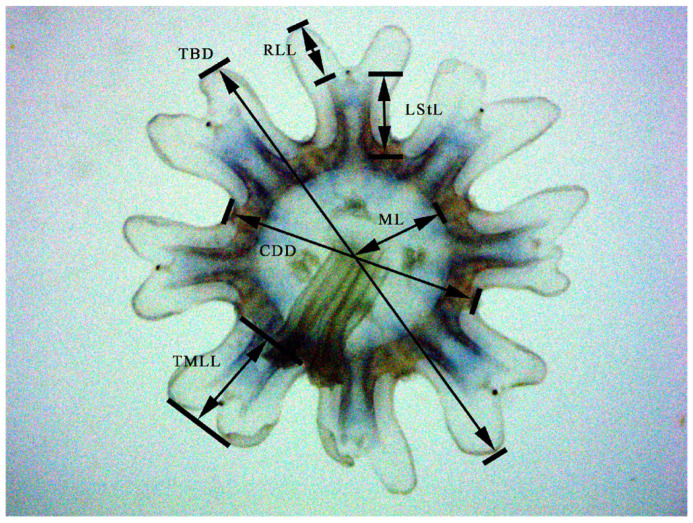
Measuring points and measurements of the ephyra of *Phyllorhiza yurena* Chen, Hu & Xing sp. nov. CDD = central disk diameter; LStL = lappet stem length; ML = manubrium length; RLL = rhopalial lappet length; TBD = total body diameter; TMLL = total marginal lappet length.

The samples were obtained from coastal waters near Ningbo (29°38′51″ N 121°46′43″ E) and Taizhou (121°37′48″ E 28°43′12″ N) in October 2018 and June 2023, respectively (Figure 1). The adult medusae were photographed in situ before sampling and dissected in the field; some tissues were stored in 75% ethanol, and the remainder were stored in a 5% formaldehyde solution. The polyps were collected and transported to the laboratory in a 1 L glass beaker at 20 ± 1 °C for 24 h in darkness. Then, the polyps were cultured in a 700 L tank at 25 ± 1 °C with dissolved oxygen > 5 ppm, pH 7.7–8.1, salinity 29.0–31.0, total ammonia < 0.05 mg/L, nitrite < 0.05 mg/L, 9 h of light and 15 h of darkness. The polyps and ephyrae were photographed via a stereoscopic microscope (Jiangnan-JSZ6S) connected to an industrial digital camera (UCMOS05100KPA) (Nanjing Jiangnan Novel Optics Co., Ltd., Nanjing, China), and the strobila, young and adult medusae were photographed with an Olympus TG5 camera (Olympus Corporation, Tokyo, Japan). Additionally, we randomly sampled and measured the dimensions of ephyrae, the measuring points contains total body diameter (TBD), central disk diameter (CDD), lappet stem length (LStL), rhopalial lappet length (RLL), manubrium length (ML) and total marginal lappet length (TMLL) of ephyrae (measured within 24 h after detachment) (see Figure 2) [13,14]. The holotype and paratype were preserved at the Yuren Museum and the Third Institute of Oceanography, MNR, China (code NB2023003—NB2023007, TIO—SRMP001, and TIO—SRMP002).

### 2.2. DNA Extraction, Amplification and Sequencing

Total genomic DNA was extracted from tissue samples of *P. yurena* Chen, Hu & Xing sp. nov. via the E.Z.N.A.R Tissue DNA Kit (Qiagen, Shanghai, China) following the manufacturer’s instructions. For phylogenetic analysis, two mitochondrial markers (cytochrome c oxidase subunit I (*COI*) and *16S* rDNA (*16S)*) and two nuclear markers (*18S* rDNA (*18S*; small subunit), *28S* rDNA (*28S*; large subunit)) were selected. A total of 25 µL of the reaction mixture was prepared for the polymerase chain reaction (PCR). The reaction mixture was composed of 2.5 µL template DNA, 12.5 µL 2× Taq Master Mix, 1 µL forward primer, 1 µL reverse primer, and 8 µL distilled water. The primers and the PCR conditions used for amplifying these sequences are provided in Table 1. The PCR result was confirmed in a 1% agarose gel via a ChemiDocTM XRS+ imaging system. Confirmed samples were selected and sent to the sequencing department of Sangon Biotechnology from Shanghai, China. PCR products were sequenced for both strands.

### 2.3. Genetic Data Analysis

The dataset for the phylogenetic analyses included the *COI, 16S, 18S*, and *28S* sequences obtained from GenBank of *Phyllorhiza* spp. excluding those unclassified species and other represented species of the family Mastigiidae (Table 2). *Lychnorhiza lucerna* Haeckel, 1880 was used as the root, following the proposal of Rosales-Catalán et al. [12]. The nucleotide sequences were aligned via MAFFT v.7 [15] with the G-INS-i algorithm. Genetic distances of *COI*, *16S*, *18S*, and *28S* between species/populations were calculated with MEGA 6.0 using the Kimura 2-parameter model [16].

The phylogenetic analyses were conducted using maximum likelihood (ML) via IQ-TREE v.2.2.0 [17] and Bayesian inference (BI) via MrBayes v.3.2.7a [18], both of which are implemented as plugins in PhyloSuite [19,20]. The ModelFinder v.2.2.0 plugin in PhyloSuite selected the best-fit model via the Bayesian Information Criterion (BIC) [21]. The optimal substitution models for the COI, 16S, 18S, and 28S alignments are detailed in Table 1. Maximum likelihood (ML) trees were inferred with 1000 standard bootstraps. Following Hillis and Bull [22], ML bootstrap values were categorized into low (<70%), moderate (70–90%), and high (≥90%) confidence levels. Bayesian inference (BI) phylogenies were reconstructed with dual parallel runs, each consisting of 10,000,000 generations and a burn-in phase that discarded the initial 25% of sampled data. The posterior probabilities were categorized as low and high for values <0.95 and ≥0.95, respectively [23].

**Table 1:** List of primers, thermocycle conditions used to amplify and the models for the maker alignments.

Markers	Primer	Sequense	PCR Conditions	Reference	Length (bp)	Model (BI)	Model (ML)
*16S*	16S-CB1	5′-TCGACTGTTTACCAAAAACATAGC-3′	33–35 cycles (94 °C for 45 s, 50–52 °C for 45 s, and 72 °C for 60 s) and 72 °C for 300 s	[24]	≈640	GTR+F+G4	TIM2+F+G4.
	16S-CB2	5′-ACGGAATGAACTCAAATCATGTAAG-3′					
*COI*	LCO1490	5′-GGTCAACAAATCATAAAGATATTGG-3′	33–35 cycles (94 °C for 45 s, 50–55 °C for 45–60 s, and 72 °C for 60 s) and 72 °C for 600 s	[25]	≈700	GTR+F+G4	TIM2+F+G4
	HCO2198	5′-TAAACTTCAGGGTGACCAAAAAATCA-3′					
	COXIF	5′-GTATTTTCTCTGGCGTACTAGGTGC-3′		present study	≈640		
	COXIR	5′-ATAAATGCTGATATAAGATGGGGTC-3′					
*28S*	Cassiopea 28S F	5’-GRCGGCGAATTGTAGTCTCGA-3’	38 cycles (94 °C for 45 s, 47–55 °C for 60–90 s, and 72 °C for 70–90 s) and 72 °C for 600 s	[13]	≈1010	GTR+F+G4	TN+F+G4
	Aa H28S 1039	5’-GTCTTTCGCCCCTATACCCA-3’					
*18S*	Aa L18S 12	5’-TCCTGCCAGTAGTCATATGCTTG-3’	38 cycles (94 °C for 45–50 s, 47–54 °C for 70 s, and 72 °C for 70–90 s) and 72 °C for 600 s	[26]	≈1770	HKY+F+I	HKY+F+I
	Aa H 18S 1798	5′-CCTACGGAAACCTTGTTACGA-3′					
	Aa H18S 1318 FC	5′-CAGACAAATCACTCCACCAAC-3′					
	Aa H18S 1318 RC	5′-GTTGGTGGAGTGATTTGTCTG-3′					

**Table 2:** List of species and accession numbers of sequences used in this study. “–” means unavailable data. Sequences of the new species are annotated in bold.

			GenBank Accession No.				
Species	Voucher/Isolate No.	Location	COI	16S	18S	28S	Reference
***Phyllorhiza yurena* sp. nov.**	**TIO-SRMP001**	**East Sea of China**	**PV366411**	**PV367414**	**PV613531**	**PV612471**	**present study**
***Phyllorhiza yurena* sp. nov.**	**TIO-SRMP002**	**East Sea of China**	**PV366405**	**PV367408**	**PV613528**	**PV612469**	**present study**
***Phyllorhiza yurena* sp. nov.**	**NB-2023003**	**East Sea of China**	**PV366406**	**PV367409**	**–**	–	**present study**
***Phyllorhiza yurena* sp. nov.**	**NB-2023004**	**East Sea of China**	**PV366408**	**PV367410**	**PV613529**	**PV612470**	**present study**
***Phyllorhiza yurena* sp. nov.**	**NB-2023005**	**East Sea of China**	**PV366409**	**PV367411**	**–**	–	**present study**
***Phyllorhiza yurena* sp. nov.**	**NB-2023006**	**East Sea of China**	**PV366407**	**PV367412**	**PV613530**	–	**present study**
***Phyllorhiza yurena* sp. nov.**	**NB-2023007**	**East Sea of China**	**PV366410**	**PV367413**	–	–	**present study**
*Phyllorhiza pacifica*	12198	Bangladesh: Teknaf	PP945789	–	–	–	Khanam et al., unpublished
*Phyllorhiza pacifica*	M0D022675C_THKRKOB	Thailand	–	KY610622	–	–	[13]
*Phyllorhiza pacifica*	M0D022673A_THKRKOP	Thailand	–	KY610623	KY610770	KY610998	[13]
*Phyllorhiza pacifica*	M0D022675C_THKRKOB	Thailand	–	–	KY610774	KY610997	[13]
*Phyllorhiza* cf. *pacifica*	M0D21426B_IDJISUY	Indonesia	MN395673	–	–	–	[27]
*Phyllorhiza punctata*	–	Israel	–	HG931681	–	–	[28]
*Phyllorhiza punctata*	NO.5-16S	Thailand	–	KT982716	–	–	[29]
*Phyllorhiza punctata*	PAZ072019_1	Mexico	MT899235	MT902932	–	–	[12]
*Phyllorhiza punctata*	PAZ072019_2	Mexico	MT904380	MT902935	–	–	[12]
*Phyllorhiza punctata*	M0D00662L	Australia	–	–	HM194770	–	[26]
*Phyllorhiza punctata*	M0D014781M_MXBSAGO	Mexico	KY611062	KY610625	KY610773	KY610999	[27]
*Phyllorhiza punctata*	M0D0147830_MXBSCPC	Mexico	KY611060	KY610626	KY610771	KY611000	[27]
*Phyllorhiza punctata*	M0D014780L_MXBSMAG	Mexico	KY611061	KY610627	KY610772	KY611001	[27]
*Phyllorhiza punctata*	M0D00662L	Australia	–	KY610624	–	HM194825	[26,27]
*Phyllorhiza punctata*	Sc18.1.1	Gulf of Mexico	GQ120101	–	–	–	[30]
*Phyllorhiza punctata*	2_S8GB	Australia	EU363342	–	–	–	[31]
*Phyllorhiza punctata*	1_S8GB	Australia	EU363341	–	–	–	[31]
*Phyllorhiza punctata*	PPMJ4	Malaysia	JN203010	JN202945	–	–	[32]
*Phyllorhiza punctata*	Phy	Malaysia	–	JN202946	–	–	[32]
*Phyllorhiza punctata*	PPKS0612	Malaysia	JN203009	–	–	–	[32]
*Phyllorhiza punctata*	PPKS0912	Malaysia	JN203008	–	–	–	[32]
*Phyllorhiza punctata*	PPKS1012	Malaysia	JN203007	–	–	–	[32]
*Phyllorhiza punctata*	PPKS0712	Malaysia	JN203006	–	–	–	[32]
*Phyllorhiza punctata*	PPKS0512	Malaysia	JN203005	–	–	–	[32]
*Phyllorhiza punctata*	PPKS0412	Malaysia	JN203004	–	–	–	[32]
*Phyllorhiza punctata*	PPKS0112	Malaysia	JN203003	–	–	–	[32]
*Phyllorhiza punctata*	PPPP04	Malaysia	JN203002	–	–	–	[32]
*Phyllorhiza punctata*	PPPP03	Malaysia	JN203001	–	–	–	[32]
*Phyllorhiza punctata*	PPPP01	Malaysia	JN203000	–	–	–	[32]
*Phyllorhiza punctata*	PPKS0110	Malaysia	JN202999	–	–	–	[32]
*Phyllorhiza punctata*	PPKS0310	Malaysia	JN202998	–	–	–	[32]
*Phyllorhiza punctata*	M0D013181Y	Australia	KU900939	KU901025	–	–	[33]
*Phyllorhiza punctata*	M0D013180X	Australia	KU900938	KU901024	–	–	[33]
*Phyllorhiza punctata*	–	Eastern Mediterranean	–	–	HG931673	HG931674	[28]
*Phyllorhiza punctata*	–	Gulf of Mexico	–	JX393272	–	–	[34]
*Phyllorhiza punctata*	–	Singapore	OR400205	OR400205	–	–	[35]
*Phyllorhiza punctata*	–	Singapore	OR400201	OR400201	–	–	[35]
*Phyllorhiza punctata*	–	Singapore	NC_084193	NC_084193	–	–	[35]
*Lychnorhiza lucerna*	M0D016088T_NIANGBW	Nicaragua	KY611034	KY610591	KY610785	KY610906	[13]
*Cassiopea frondosa*	M0D021382J_PABTBDE	Panama	KY610557	KY610615	KY610767	KY611004	[13]
*Cassiopea andromeda*	M0D006024R_MXBCISJ	Mexico	KY610551	KY610609	KY610763	KY611005	[13]
*Mastigias papua*	M0D06000T	Palau	–	–	HM194796	HM194849	[33]
*Mastigias papua*	M0D005915M	Palau	KU901397	KU901021	–	–	[33]
*Versuriga anadyomene*	M0D00095Q	Palau	–	–	HM194768	HM194823	[33]
*Versuriga anadyomene*	-	South China Sea	KX904853	KX904852	–	–	[36]

## 3. Results

### 3.1. Systematics

Class Scyphozoa Goette, 1887

Subclass Discomedusae Haeckel, 1880

Order Rhizostomeae Cuvier, 1800

Family Mastigiidae Stiasny, 1920

Genus *Phyllorhiza* Agassiz, 1862

**Figure 3:**
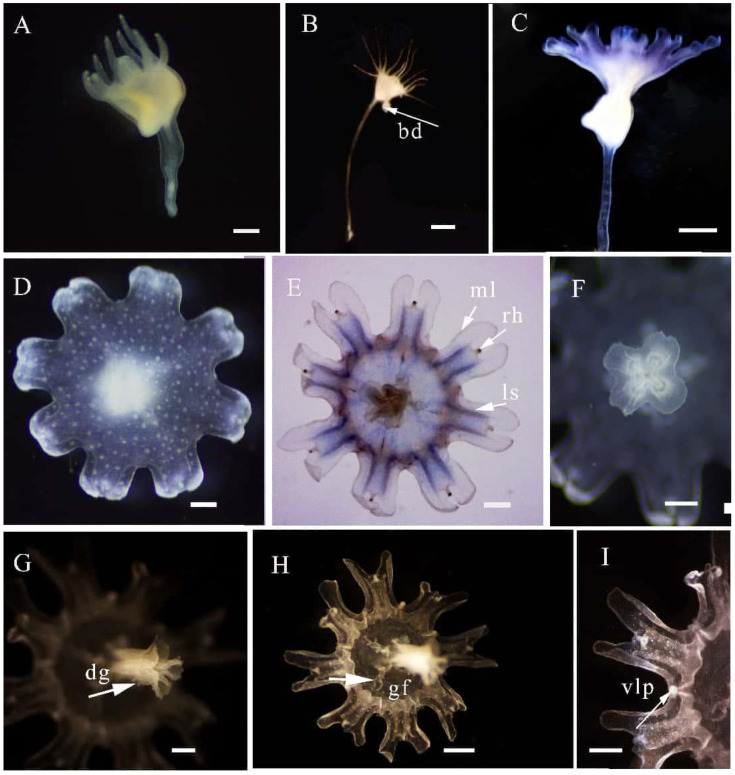
Morphological characteristics of polyp, strobilation, and ephyra stages of *Phyllorhiza yurena* Chen, Hu & Xing sp. nov. Holotype NB2023003. ((**A**–**F**) alive; (**G**–**I**) preserved). (**A**) lateral view of polyp; (**B**) view of a polyp with planuloid budding; (**C**) view of strobila; (**D**) aboral view of ephyra; (**E**–**I**) view of ephyra; (bd = bad; ml = marginal lappets; rh = rhopalium; ls = lappet stems; gf: gastric filaments; dg = digitata; vlp = velar lappets). Scale bars: (**A**,**C**,**D**) = 0.2 mm; (**E**–**I**) = 0.5 mm; (**B**) = 1.0 mm.

**Figure 4:**
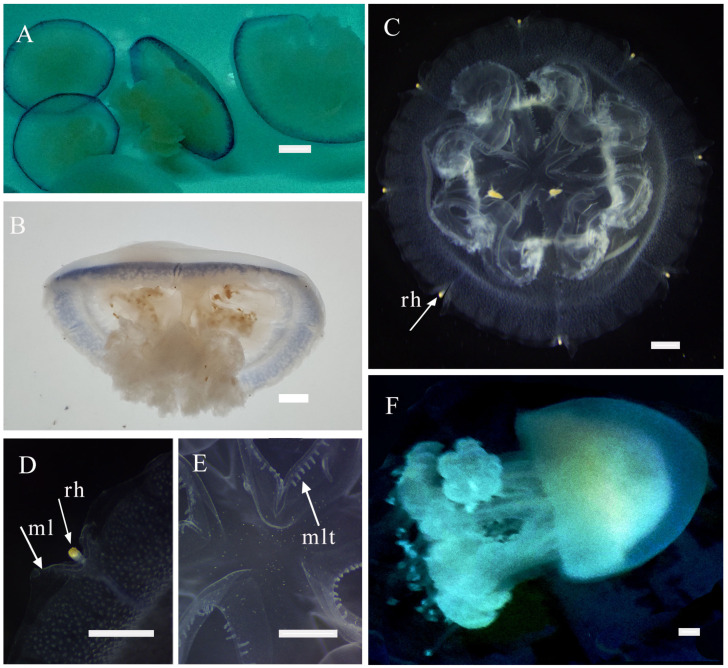
Morphological characteristics of medusa *Phyllorhiza yurena* Chen, Hu & Xing sp. nov. Holotype NB2023003. (**A**) young medusae with a blue ring; (**B**) lateral view of a young medusa; (**C**) oral view of a young medusa, (**D**) rhopalium; (**E**) closure of the central mouth; (**F**) adult medusa bred in the lab. (ml = marginal lappets; rh = rhopalium; mlt = mouthlets). Scale bars: (**A**,**B**) = 0.5 cm; (**C**–**E**) = 1 cm; (**F**) = 1.5 cm.

**Figure 5:**
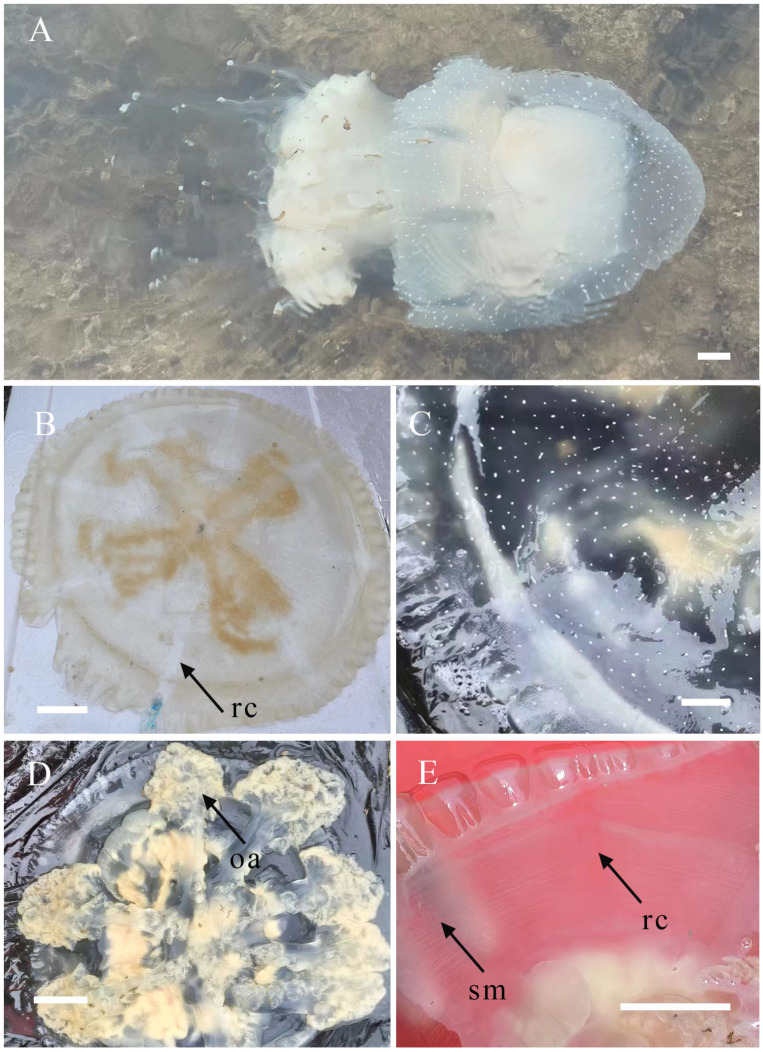
Morphological characteristics of medusa *Phyllorhiza yurena* Chen, Hu & Xing sp. nov. Paratype TIO-SRMP001 (wild-collected adult specimen). (**A**) lateral view of adult medusa; (**B**) disk of medusa; (**C**) spots on the disk of medusa; (**D**) oral view of medusa; (**E**) canal of medusa. (rc = radial canal; oa = oral arms; sm = striated muscles). Scale bars: (**A**,**C**,**E**) = 2.5 cm; (**B**,**D**) = 5.0 cm.

*Phyllorhiza yurena* Chen, Hu & Xing sp. nov. (Figure 3, Figure 4 and Figure 5)

LSIDurn:lsid:zoobank.org:act:BDFF267C-EAC4-46DE-A2BB-08F628994827

**Material examined.** Holotype (NB2023003) and paratype (NB2023004–NB2023007) were collected polyps from coastal waters near Ningbo (29°38′51″ N 121°46′43″ E) in October 2018 and June 2023, then bred at Ningbo Ocean World Laboratory. Paratype (TIO-SRMP001 and TIO-SRMP002) was obtained adult medusae from coastal waters near Taizhou (121°37′48″ E 28°43′12″ N) in June 2023.

**Etymology.** The species name is from the Latin Yurena, meaning Yuren, which refers to the Yuren Marine Industry Development Company for its support for the jellyfish research in recent years.

**Type locality.** East China Sea.

**Diagnosis.** The polyp is triangular or square with 14–17 tentacles, and the base is root-like. The lappet stems of ephyra are blue, the 16 marginal lappets are transparent, and the aboral surface is covered with warts; a blue ring encircles the umbrella edge of the young medusa, when the umbrella diameter increases to approximately 3–5 cm, the blue ring gradually disappears; the adult body is white, with more than one club-shaped appendage with a distal swelling on each mouth arm. There are 8 radial canals in the subumbrella, and 13–17 inter rhopaliar radial canals at per octant eight. Two smaller rhopaliar lappets and 9–16 bigger velar lappets at per octant eight. Gastrovascular system consisting of a central stomach with eight radial rhopaliar canals, a ring canal present, and developed striated muscles. The adult medusa is up to 250~450 mm wide and has a hemispherical umbrella; exumbrella with white spots and warts, with numerous short, club-shaped filament appendages on the lower parts of the mouth arms; the mouth arms are three-winged, and mouthlets occupy two-thirds of the length of the mouth arms.

**Description.** The calyx of the polyp is square, and the thin stalk is approximately eight times as long as the calyx. There are sixteen tentacles around the calyx edge. The base is root-like, and the polyp is white. There are tentacle-like organs under the mouth of the ephyra, four groups of gastric filaments around the mouth, and wart-like protrusions on the anti-oral surface. Larvae have four body cavities and small mouths that gradually grow from inside the oral arms.

**Polyp stage.** When the polyp of the new species is fully extended, the hypostome is triangular or square (Figure 3A,B), there are 14–17 tentacles around the hypostome edge, thin, elongate stalks at the basal end, and the base is root-like. In general, the length of a stalk is approximately two–four times greater than that of a hypostome; however, after feeding, the stalk continues to extend, and the length of the stalk is approximately six–eight times greater than that of the calyx (Figure 3B). The main method of asexual reproduction is planuloid budding (Figure 3B). The bud can swim in sea water, and one set becomes another polyp sometime later. The polyp is white when no food is offered. The above characteristics are consistent between the holotype and paratypes.

**Strobilation and Ephyra stage.** During strobilation, the tentacles of the polyp disappear; later, they become strobila, and they have monodisc strobilation, with a single ephyra forming at one time. The ephyrae have eight pairs of marginal lappets, eight rhopalia, a central mouth, four gastric cavities, and one or two gastric filaments in each cavity, and the exumbrella is covered with warts (Figure 3C,D). It has a cruciform mouth and fine finger-like projections on the oral arms and the digitata. The lappet stems of the new species are blue, and the marginal lappets are transparent (Figure 3E–H). The ratios of dimensions of ephyrae are shown in Table 3. Velar lappets appear gradually over time (Figure 3I). The above characteristics are consistent between the holotype and paratypes.

**Young medusae.** With the growth of jellyfish, the marginal lappets of new species gradually develop and form a complete umbrella, the manubrium is small, the mouthlets gradually develop on the inside of the mouth arms, and the terminal club-shaped appendage slowly grows with distal swelling (Figure 4A,B). When the umbrella diameter increases to approximately 3–5 cm, the blue ring gradually disappears, and the aboral surface is covered with warts (Figure 4C,D). The primary oral mouth closed (Figure 4E). The above characteristics are consistent between the holotype and paratypes.

**Adult medusa.** Later in development, warts emerge on the exumbrella, forming white spots (Figure 4F). The characteristics of the adult medusa are consistent between the holotype and paratypes. The most significant differences are in coloration and adult size between wild-collected and lab-bred specimens. Under the feeding conditions, their umbrella becomes brown due to the proliferation of zooxanthellae, and the diameter of the jellyfish umbrella increases to 200–250 mm in the laboratory (Figure 4F), while the wild-collected ones reach up to 450 mm in diameter (Figure 5A). The adult has a hemispherical umbrella and mouth arms, and they are milky and white. There are 8 radial canals in the subumbrella, and 13–17 inter rhopaliar radial canals at per octant eight. Two smaller rhopaliar lappets and 9–16 bigger velar lappets at per octant eight. The exumbrella has white warts on the central part extending to the umbrella margin, but the edge warts are not larger or numerous (Figure 5B,C). There is more than one club-shaped appendage with a distal swelling on each mouth arm. The mouth arms are three-winged, and the mouthlets occupy two-thirds of the length of the mouth arms (Figure 5D). Gastrovascular system consisting of a central stomach with eight radial rhopaliar canals, ring canal present. No observation of additional radial canals. No radial muscles, developed striated muscles (Figure 5E).

**Remark.** *Phyllorhiza yurena* Chen, Hu & Xing sp. nov. is assigned to the genus *Phyllorhiza* based on its morphological characteristics and molecular phylogenetic analyses. The new species has short, pyramidal, three-winged mouth arms, with numerous filaments on the lower parts of the mouth arms. These characteristics are consistent with the general characteristics of *Phyllorhiza*. However, the new species can be distinguished from other species within the genus (two valid species and two uncertain species recorded by WoRMS [37]) by the following unique features: (1) The exumbrella of *P. yurena* Chen, Hu & Xing sp. nov. is covered with white warts that extend from the central part to the margin of the umbrella. These warts are distinct from the granular or tuberculated textures found in other species such as *P. chinensis* and *P. trifolium*. (2) The lower parts of the mouth arms in *P. yurena* Chen, Hu & Xing sp. nov. have numerous short, club-shaped filament appendages with distal swellings. This is in contrast to species like *P. punctata*, which have fewer and longer appendages. (3) The overall coloration of *P. yurena* Chen, Hu & Xing sp. nov. is milky white, with a distinctive blue annular band circumscribing the umbrella margin in young medusae. This is different from the brownish or greenish hues observed in other species such as *P. pacifica* and *P. luzoni*. (4) The mouth arms of *P. yurena* Chen, Hu & Xing sp. nov. are three-winged, and the mouthlets occupy approximately two-thirds of the length of the mouth arms. This is a unique feature that sets it apart from other species within the genus.

### 3.2. Genetic Distance and Phylogenetic Analyses

**Figure 6:**
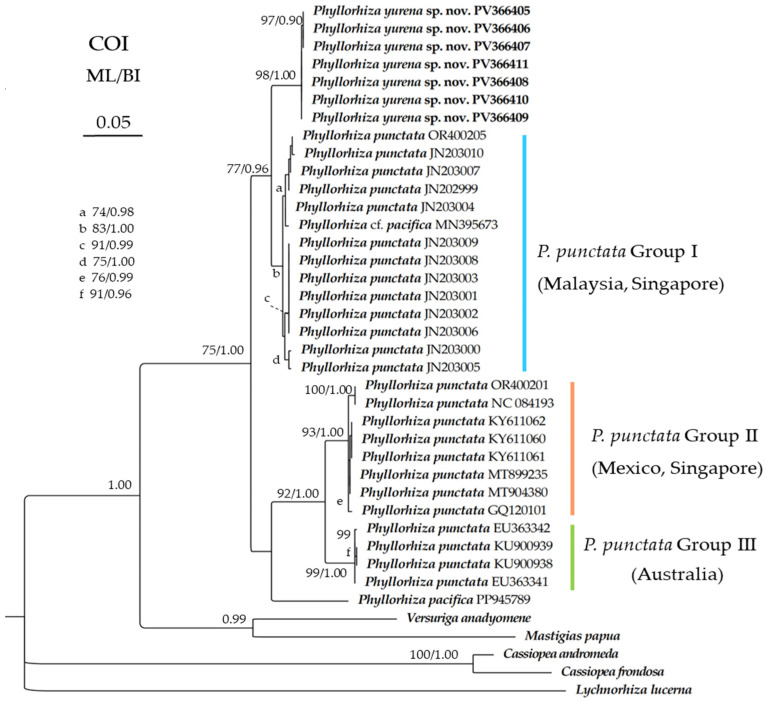
Maximum likelihood (ML) tree inferred from the *COI* sequences of Mastigiidae. The Bayesian inference (BI) tree is identical to the ML tree in topology. Node support is as follows: ML bootstrap/BI posterior probability. Only values with ML bootstrap ≥70% or BI posterior probability ≥0.90 are shown. Sequences of the new species are annotated in bold.

**Figure 7:**
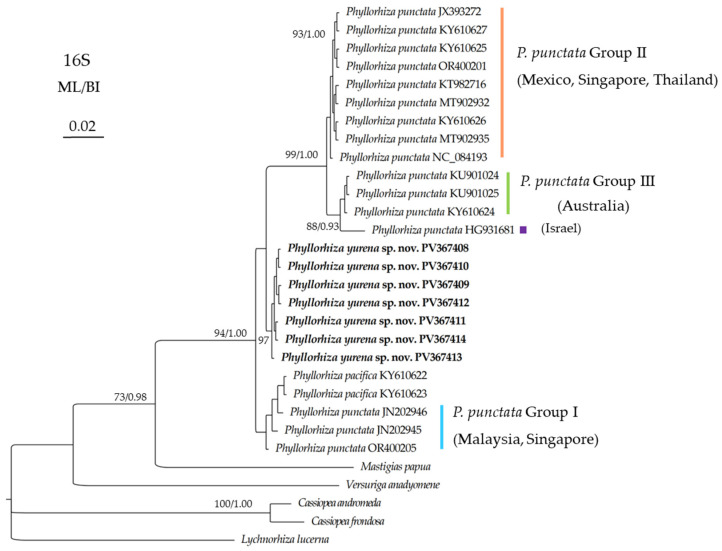
Maximum likelihood (ML) tree inferred from the *16S* sequences of Mastigiidae. The Bayesian inference (BI) tree is identical to the ML tree in topology. Node support is as follows: ML bootstrap/BI posterior probability. Only values with ML bootstrap ≥70% or BI posterior probability ≥0.90 are shown. Sequences of the new species are annotated in bold.

**Figure 8:**
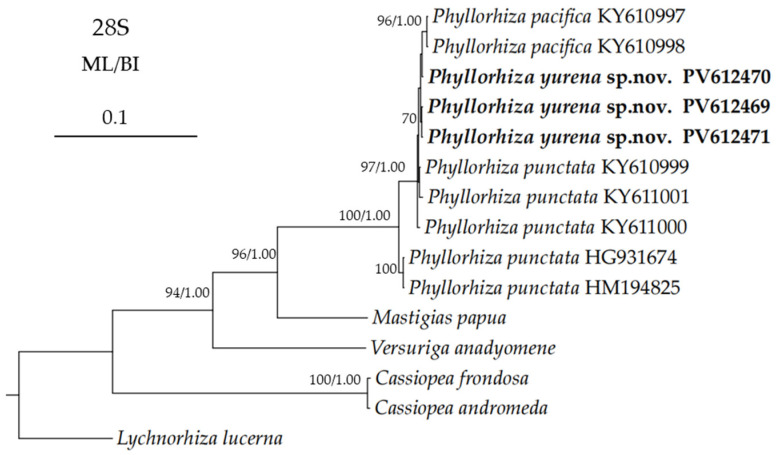
Maximum likelihood (ML) tree inferred from the *28S* sequences of Mastigiidae. The Bayesian inference (BI) tree is identical to the ML tree in topology. Node support is as follows: ML bootstrap/BI posterior probability. Only values with ML bootstrap ≥70% or BI posterior probability ≥0.90 are shown. Sequences of the new species are annotated in bold.

**Figure 9:**
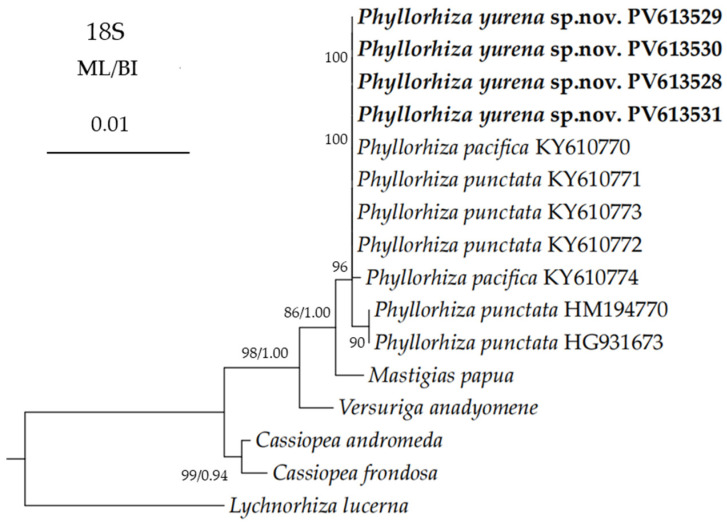
Maximum likelihood (ML) tree inferred from the *18S* sequences of Mastigiidae. The Bayesian inference (BI) tree is identical to the ML tree in topology. Node support is as follows: ML bootstrap/BI posterior probability. Only values with ML bootstrap ≥70% or BI posterior probability ≥0.90 are shown. Sequences of the new species are annotated in bold.

The newly obtained sequences were deposited in GenBank (Table 1). The alignments comprised 1779, 1228, 663, and 600 nucleotide positions for the *18S*, *28S*, *COI*, and *16S* regions, respectively. The maximum likelihood (ML) tree is nearly identical to the Bayesian inference (BI) tree in topology for all the regions, and thus a single tree with both support values was shown for each region (Figure 6, Figure 7, Figure 8 and Figure 9). In the *COI* trees, all the *Phyllorhiza* species formed a monophyletic clade, and all the populations of *Phyllorhiza punctata* were separated into three groups (Groups I, II, and III; Figure 6). *Phyllorhiza yurena* sp. nov. formed a subclade with *P. punctata* Group I with moderate to high support (ML 77%; BI 0.96), while *P. pacifica* clustered with *P. punctata* Groups II + III with high support (ML 92%; BI 1.00). Based on the *COI* aligned region, the intraspecific distances of the new species ranged from zero to 0.2%. The distances between the new species and *P. punctata* Group I were in the range of 1.62–2.44%, while the distances between the new species and other *Phyllorhiza* species/groups were in the range of 8.78–10.24% (Table 4).

In the *16S* trees, all the *Phyllorhiza* species formed a monophyletic clade (Figure 7). All the populations of *Phyllorhiza punctata* were separated into four subclades (Group I, II, III, and *P. punctata* HG931681). *Phyllorhiza yurena* sp. nov. clustered with the subclade including *P. punctata* Groups II + III + HG931681 with low support (ML < 70%; BI < 0.90). Based on the *16S* aligned region, the intraspecific distances of the new species is zero, the distances between the new species and *P. punctata* Group I were in range of 0.40–0.68%, while the distances between the new species and other *Phyllorhiza* species/groups were in the range of 1.22–4.72% (Table 5).

Based on the 28S aligned regions, the interspecific distances among *Phyllorhiza* spp. were relatively low (0–1.62%), the new species showed little variation compared to *P. pacifica* and the Mexican population of *P. punctata*. However, the distances between the new species and the Australian and Eastern Mediterranean populations of *P. punctata* were in the range of 1.31–1.49% (Table 6). In the *28S* trees, the new species was nested within the *Phyllorhiza* clade, but its precise phylogenetic position could not be resolved due to the low support values (Figure 8).

Based on the *18S* aligned regions, the genetic distances among *Phyllorhiza* spp. were extremely low, with values ranging from only zero to 0.12% (Table 7). Similarly to the *28S* trees, the new species was nested within the *Phyllorhiza* clade, but its phylogenetic position remained unresolved due to little variation among the congeners (Figure 9).

## 4. Discussion

### 4.1. Morphological Differences Between the New Species and Congeners

The morphological characteristics of different life cycle stages of the newly described species *P. yurena* Chen, Hu & Xing sp. nov. were meticulously observed and compared with those of other *Phyllorhiza* species. The results revealed that the adult medusae of *P. yurena* exhibit distinct differences from those of other *Phyllorhiza* species. Moreover, significant variations were also observed among the different life cycle stages of *P. yurena* itself, as well as in the distribution patterns of these stages. In general, *P. yurena* Chen, Hu & Xing sp. nov. exhibits distinct differences in umbrella shape, color, appendage morphology and number, and distribution compared to other recorded *Phyllorhiza* species.

*Phyllorhiza pacifica*, predominantly found in the Philippines [38,39], is characterized by its flat-hemispherical umbrella and an exumbrella adorned with a myriad tiny brown spots. The presence of zooxanthellae causes these spots to become more irregular as they approach the edge of the umbrella, often appearing larger and more abundant in number. With an umbrella diameter ranging from 20 to 30 mm, which exhibits a brownish hue, and the clubs are tinged with purple. In contrast, adult *P. yurena* Chen, Hu & Xing sp. nov. boasts a milky white color, with the exumbrella featuring white warts that spread from the center to the margin of the umbrella, yet the spots at the edge are neither large nor numerous. At an umbrella diameter of 20 to 30 mm, the medusa is white with a distinctive blue annular band circumscribing the umbrella margin, and the appendages are consistently white, setting it apart from *P. pacifica*.

*Phyllorhiza luzoni* stands out from its congeners due to its broad, flat exumbrella featuring a delicately granular texture. The exumbrella is dotted with white spots that are more irregular, numerous, and larger towards the periphery than at the center. This species has a green hue and possesses small appendages on its mouth arms. *Phyllorhiza luzoni* is native to Varadero Bay in southern Luzon and the Philippine islands [40,41]. In contrast, the new species is distinguished by its hemispherical umbrella and pristine white coloration, and it was found in the East China Sea.

**Figure 10:**
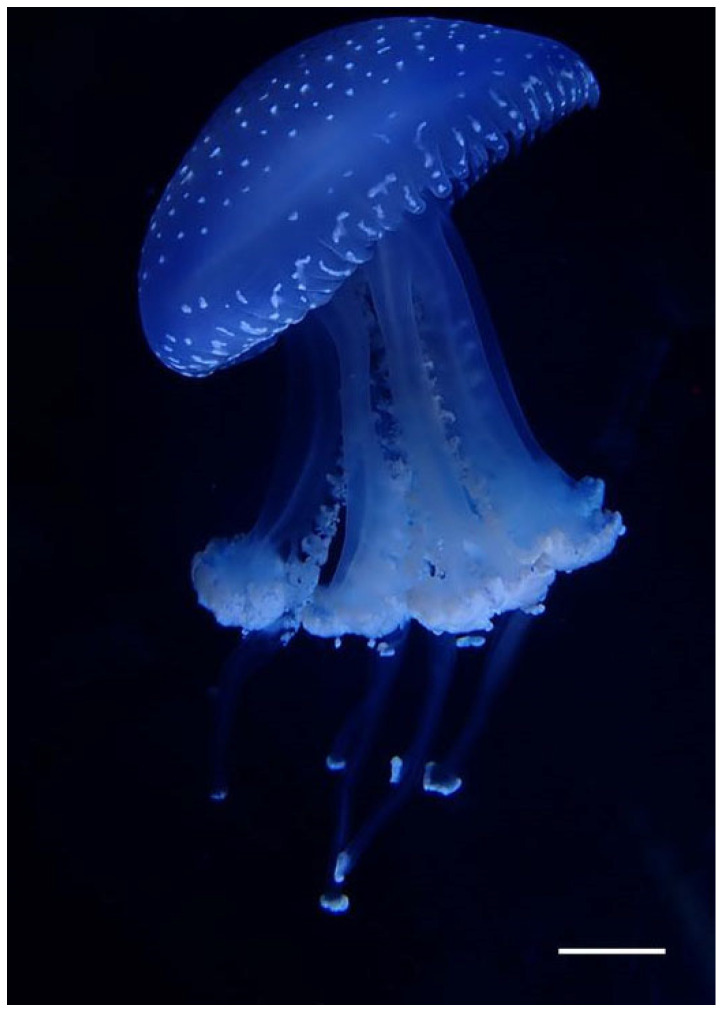
*Phyllorhiza punctata* von Lendenfeld, 1884 (laboratory-cultured adult specimen) Photoed by Zhijie Hu. Scale bar = 5.0 cm.

*Phyllorhiza punctata* has a hemispherical umbrella with obvious warts, with bumpy surface, and one main club-shaped appendage with distal swelling on every mouth arm, as described by Mayer [42] and Arsiranant et al. [43] (Figure 10). These features are most similar to those of the new species. However, *P. yurena* Chen, Hu & Xing sp. nov. has more than one club-shaped appendage on every mouth arm. During growth, the calyx of the polyp of new species has been observed to be square and triangular, whereas *P. punctata*’s calyx is only square. Additionally, the ephyra of the new species has blue lappet stems with transparent marginal lappets, while *P. punctata* has a brown appearance with smooth margins. Furthermore, the medusa of the new species reaches a maximum width of 450 mm, whereas *P. punctata* can expand up to 600 mm in width.

Although the species *P. chinensis* L. Agassiz, 1862 and *P. trifolium* Haeckel, 1880 were imperfectly described, as stated by Mayer [42], there are still obvious differences from our new species. The disk of *P. chinensis* is papillous or tuberculated, and the tubercles are larger toward the summit of the umbrella. The marginal lappets are reddish-brown, marked by darker streaks, and this species is found in the China Seas [44]. In contrast, the ephyra lappet stems of *P. yurena* Chen, Hu & Xing sp. nov. are blue, and its marginal lappets are transparent, with no darker streaks observed. As our new species grows, its spots become irregular, but they do not increase in size towards the umbrella’s summit. *P. trifolium* bears a resemblance to *P. chinensis*, but it is distinguished by its 24 very long filaments and is found in the Japan Sea [45].

### 4.2. DNA Barcoding Analysis of Phyllorhiza

DNA barcoding, utilizing mitochondrial and/or nuclear markers, is widely recognized as a crucial initial step in the integrative identification of jellyfish species [13,27,30,32,33]. In our analysis, the nuclear *18S* region exhibited significantly less variation compared to the other three loci, with minimal genetic variability observed among *Phyllorhiza* spp. (Table 7). Consequently, the *18S* marker is deemed less informative for species-specific identification within jellyfish. For the *28S* marker, no barcoding gap was detected for delimitating *Phyllorhiza* species, as no genetic variability was observed between *P. yurena* sp. nov. and the Mexican population of *P. punctata* (Table 6). This highlights the limited utility of *28S* for species delimitation within this genus. In contrast, the mitochondrial *COI* and *16S* markers displayed a higher level of genetic variation within the genus *Phyllorhiza* (Table 4 and Table 5).

In a comprehensive DNA barcoding study by Ortman et al. [30], intraspecific *COI* distances ranged from zero to 5.7% (mean 1.3%), while distances between congeneric species ranged from 5.6% to 38.1% for Medusozoa. In our study, the *COI* genetic distances between *P. punctata* Group I and Groups II + III were between 8.78% and 11.46%, suggesting that these groups represent distinct species (Table 4). Given that *P. punctata* was originally described from Australia [46], which is the same location as Group III, it is plausible that Group I may not be the true *P. punctata*.

The *16S* marker appears to mirror the *COI* gene in terms of genetic distance patterns (Table 5). However, due to the lack of detailed morphological data, the identification of Group I remains uncertain. Moreover, effective genetic thresholds for *COI* and *16S* have yet to be determined for the genus *Phyllorhiza*. Therefore, to optimize the DNA barcoding framework for *Phyllorhiza*, additional data, including both conspecific and congeneric sequences with accurate morphological identification, are urgently needed.

### 4.3. Key to the Species of the Genus Phyllorhiza

The new species, *Phyllorhiza yurena* Chen, Hu & Xing sp. nov, which is found in coastal waters and exhibits the Scyphozoa ecological characteristics. It primarily feeds on zooplankton and plays a role as a predator, and contributes to the energy flow and nutrient cycling in its habitat. The frequent occurrence of this species highlights the diversity and complexity of marine ecosystems and underscores the need for further ecological studies to understand its interactions with other species. Based on this study, a dichotomous key for the existing species of this genus has been developed, which is expected to provide a foundation for further research.

1. Umbrella broad and flat, mouth arms slender, strongly compressed ……………………………………………………………………………………………………………‥*Phyllorhiza luzoni* Mayer, 1915

- Umbrella hemispherical ……………………………………………………………………………………………………………………………………………………………………………………………………2

2. Marginal lappets marked darker streaks, the disk is papillous or tuberculated, and the tubercles are larger toward the summit of the umbrella ………………………………………………………………………………………………………………………………………………………………………‥‥‥.*Phyllorhiza chinensis* L. Agassiz, 1862

- Marginal lappets without darker streaks …………………………………………………………………………………………………………………………………………………………………………………3

3. Marginal lappets with 24 very long filaments …………………………………………………………………………………………………………………………………‥*Phyllorhiza trifolium* Haeckel, 1880

- Marginal lappets without filaments ………………………………………………………………………………………………………………………………………………………………………………………4

4. Terminal appendages with a distal swelling ……………………………………………………………………………………………………………………………………………………………………………5

- Terminal appendages without a distal swelling, umbrella flat-hemispherical and up to 400 mm wide, exumbrella with numerous minute brown spots, terminal appendages nearly as long as the mouth arms; predominantly purple ………………………………………………………………………………………………………………………………………………*Phyllorhiza pacifica* (Light, 1921)

5. Umbrella up to 600 mm wide, jelly very thick, exumbrella with finely granular surface; marginal lappets some broad and double, others simple, altogether up to 14 in each octant; arm-disk with numerous filaments; lower parts of mouth arms with a terminal white club-shaped appendage, some of which up to two-thirds as long as the mouth arms themselves …………………………………………………………………………………………………………………………………………………………………‥*Phyllorhiza punctata* von Lendenfeld, 1884

- Umbrella up to 450 mm wide, exumbrella with white spots and warts; with numerous short, club-shaped filamentous appendages on the lower parts of each mouth arm; mouth arms three-winged, and mouthlets occupy two-thirds the length of mouth arms ……………………………………………………………………………………………………………………………………………………………………….*Phyllorhiza yurena* Chen, Hu & Xing sp. nov.

## 5. Conclusions

The discovery of our new species highlights the importance of integrative taxonomy in accurately identifying and classifying jellyfish species. Both laboratory-cultured and wild-collected specimens were confirmed to belong to the same new species through morphological evidence and genetic data. This study not only establishes *Phyllorhiz yurena* sp. nov. as a distinct species but also enriches the genetic information available for the genus *Phyllorhiza*. The comprehensive identification key provided will aid future research and management efforts.

## Figures and Tables

**Table 3:** Ratios of actual measurements for ephyrae.

Body Proportions	Results
CDD/TBD	49.4~55.9%
TMLL/TBD	20.9~26.7%
RLL/TMLL	32.3~42.8%
LStL/TMLL	57.2~67.7%
ML/CDD	44.1~48.5%

CDD, central disk diameter. TBD, total body diameter. TMLL, total marginal lappet length. RLL, rhopalial lappet length. LStL, lappet stem length. ML, manubrium length.

**Table 4:** Interspecific and intraspecific distances at *COI* of *Phyllorhiza* and closely related species.

	Species/Population	Location	1	2	3	4	5	6	7	8	9	10	11	12	13	14	15	16	17	18	19	20	21	22
1	***Phyllorhiza yurena* sp. nov. PV366405-7**	East China Sea	0																					
2	***P. yurena*** **sp. nov. PV366408-11**	East China Sea	0.20%	0																				
3	*Phyllorhiza* cf. pacifica MN395673	Indonesia	2.24%	2.03%	-																			
4	*P. punctata* JN203007	Malaysia	2.03%	1.82%	0.60%	-				***P. punctata* Group I**														
5	*P. punctata* JN203004	Malaysia	2.03%	1.82%	0.20%	0.40%	-																	
6	*P. punctata* JN203010, OR400205	Malaysia, Singapore	2.24%	2.03%	0.80%	0.20%	0.60%	0																
7	*P. punctata* JN203001-3,6,8,9	Malaysia	2.24%	2.03%	0.80%	1.00%	0.60%	1.21%	0															
8	*P. punctata* JN202999	Malaysia	1.82%	1.62%	0.40%	0.20%	0.20%	0.40%	0.80%	-														
9	*P. punctata* JN203000	Malaysia	2.44%	2.24%	1.00%	1.21%	0.80%	1.41%	0.60%	1.00%	-													
10	*P. punctata* JN203005	Malaysia	2.24%	2.03%	1.21%	1.41%	1.00%	1.62%	0.80%	1.21%	0.20%	-												
11	*P. punctata* OR400201, NC_084193	Singapore	10.00%	10.24%	11.21%	10.97%	10.97%	10.97%	10.72%	10.72%	11.46%	11.21%	-	**Group II**										
12	*P. punctata* KY611060-2, MT899235, MT904380	Mexico	9.74%	9.98%	10.95%	10.70%	10.70%	10.70%	10.46%	10.46%	11.19%	10.95%	1.00%	0										
13	*P. punctata* GQ120101	Mexico	9.52%	9.76%	10.72%	10.48%	10.48%	10.48%	10.24%	10.24%	10.97%	10.72%	0.80%	0.20%	-									
14	*P. punctata* EU363342	Australia	9.01%	9.25%	10.20%	9.96%	9.96%	9.96%	10.20%	9.72%	10.44%	10.20%	4.99%	4.77%	4.56%	-	**Group III**							
15	*P. punctata* EU363341	Australia	8.78%	9.01%	9.96%	9.72%	9.72%	9.72%	9.96%	9.48%	10.20%	9.96%	4.78%	4.55%	4.34%	0.20%	-							
16	*P. punctata* KU900938, KU900939	Australia	9.01%	9.25%	10.20%	9.96%	9.96%	9.96%	10.20%	9.72%	10.44%	10.20%	4.99%	4.77%	4.56%	0.40%	0.20%	0						
17	*P. pacifica* PP945789	Bangladesh	10.46%	10.22%	11.19%	11.94%	11.44%	11.69%	11.69%	11.69%	11.94%	11.69%	11.91%	11.64%	11.42%	11.38%	11.13%	10.89%	-					
18	*Mastigias papua*	Palau	21.71%	21.71%	22.27%	22.27%	21.99%	22.27%	22.84%	21.99%	21.99%	21.71%	23.56%	23.24%	22.98%	22.40%	22.11%	20.64%	23.82%	-				
19	*Versuriga anadyomene*	Palau	19.48%	19.21%	20.28%	20.55%	20.28%	20.55%	20.55%	20.28%	20.83%	20.55%	21.19%	20.62%	20.37%	20.64%	20.37%	22.40%	18.18%	18.73%	-			
20	*Cassiopea andromeda*	Mexico	22.14%	22.14%	22.14%	22.70%	22.42%	22.98%	21.86%	22.42%	22.42%	22.70%	22.96%	23.51%	23.25%	23.86%	23.57%	23.86%	26.14%	27.97%	23.50%	-		
21	*Cassiopea frondosa*	Panama	23.53%	23.53%	24.10%	24.10%	23.82%	24.39%	23.82%	23.82%	23.82%	23.53%	25.21%	25.77%	25.50%	24.37%	24.09%	24.37%	26.66%	28.50%	24.91%	7.68%	-	
22	*Lychnorhiza lucerna*	Nicaragua	28.35%	28.35%	29.27%	29.58%	29.27%	29.90%	28.96%	29.27%	28.35%	28.65%	31.80%	33.08%	32.79%	29.87%	29.56%	29.56%	28.44%	28.96%	29.70%	28.37%	27.40%	-

Different colors represent phylogenetic groups: Blue: *P. punctata* Group I; Orange: *P. punctata* group II, Green: *P. punctata* group III. Sequences of the new species are annotated in bold.

**Table 5:** Interspecific and intraspecific distances at *16S* of *Phyllorhiza* and closely related species.

	Species/Population	Location	1	2	3	4	5	6	7	8	10	11	12	13	14	15	16	17	18
1	***Phyllorhiza yurena* sp. nov. PV367408-14**	East China Sea	0																
2	*P. pacifica* KY610622, KY610623	Thailand	1.22%	0															
3	*P. punctata* JN202945 *, JN202946 *	Malaysia	0.40%	0	0		***P. punctata* Group I**												
4	*P. punctata* OR400205	Singapore	0.68%	0.52%	0	-													
5	*P. punctata* KT982716 **	Thailand	4.68%	5.01%	-	4.99%	-												
6	*P. punctata* KY610625-7	Mexico	3.37%	3.92%	2.09%	3.74%	0	0			**Group II**								
7	*P. punctata* JX393272	Gulf of Mexico	3.55%	3.93%	2.11%	3.92%	0	0	-										
8	*P. punctata* OR400201, NC_084193	Singapore	3.49%	3.92%	2.02%	3.85%	0	0	0	0									
10	*P. punctata* MT902932, MT902935	Mexico	3.59%	3.99%	1.87%	3.98%	0	0	0	0	0								
11	*P. punctata* KU901024-5	Australia	4.28%	4.67%	2.52%	4.65%	0.85%	0.69%	0.70%	0.69%	0.74%	0		**Group III**					
12	*P. punctata* KY610624	Australia	4.11%	4.67%	2.52%	4.48%	0.86%	0.69%	0.70%	0.69%	0.74%	0	-						
13	*P. punctata* HG931681	Israel	4.72%	6.00%	2.60%	5.34%	2.90%	1.74%	2.03%	2.03%	2.03%	1.44%	1.15%	-					
14	*Mastigias papua*	Palau	13.26%	13.26%	8.29%	13.47%	17.97%	14.31%	14.36%	14.31%	13.32%	14.53%	14.53%	17.00%	-				
15	*Lychnorhiza lucerna*	Nicaragua	17.79%	17.79%	11.66%	18.02%	22.44%	18.66%	18.74%	18.66%	18.13%	18.66%	18.66%	24.24%	20.28%	-			
16	*Cassiopea andromeda*	Mexico	20.52%	20.56%	10.00%	21.00%	29.32%	21.72%	21.91%	21.91%	21.20%	22.39%	22.21%	24.49%	19.41%	19.23%	-		
17	*Cassiopea frondosa*	Panama	20.52%	20.56%	10.99%	20.52%	28.44%	21.72%	21.91%	21.91%	21.20%	22.39%	22.21%	24.09%	18.94%	20.43%	2.89%	-	
18	*Versuriga anadyomene*	South China Sea	17.97%	17.18%	8.74%	17.97%	23.38%	18.71%	18.71%	18.71%	18.71%	19.52%	19.52%	21.48%	20.65%	21.39%	20.88%	21.43%	-

*, indicates the sequence length is only 254 bp. **, marks indicate the sequence length is only 354 bp. Different colors represent phylogenetic groups: Blue: *P. punctata* Group I; Orange: *P. punctata* group II, Green: *P. punctata* group III. Sequences of the new species are annotated in bold.

**Table 6:** Interspecific and intraspecific distances at *28S* of *Phyllorhiza* and closely related species.

	Species/Population	Location	1	2	3	4	5	6	7	8	9	10	11
**1**	***Phyllorhiza yurena* sp. nov. PV612469-71**	East China Sea	0										
2	*Phyllorhiza punctata* KY610999	Mexico	0	0									
3	*Phyllorhiza pacifica* KY610997, KY610998	Thailand	0.10%	0.09%	0								
4	*Phyllorhiza punctata* KY611000	Mexico	0.10%	0.09%	0.43%	-							
5	*Phyllorhiza punctata* KY611001	Mexico	0.10%	0.09%	0.52%	0.26%	-						
6	*Phyllorhiza punctata* HM194825, HG931674	Australia/Eastern Mediterranean	1.62%	1.31%	1.67%	1.37%	1.47%	0					
7	*Mastigias papua* HM194849	Palau	12.16%	10.80%	11.22%	11.10%	10.99%	11.43%	-				
8	*Versuriga anadyomene* HM194823	Palau	16.47%	13.85%	14.39%	14.06%	14.17%	16.06%	12.74%	-			
9	*Cassiopea frondosa* KY611004	Panama	20.88%	17.43%	17.62%	17.47%	17.60%	20.13%	18.41%	17.05%	-		
10	*Cassiopea andromeda* KY611005	Mexico	20.88%	17.78%	17.80%	17.91%	17.91%	20.83%	19.09%	17.47%	0.19%	-	
11	*Lychnorhiza lucerna* KY610906	Nicaragua	17.49%	15.37%	15.74%	15.74%	15.74%	17.51%	15.39%	14.46%	15.25%	15.06%	-

Sequences of the new species are annotated in bold.

**Table 7:** Interspecific and intraspecific distances at *18S* of *Phyllorhiza* and closely related species.

	Species/Population	Location	1	2	3	4	5	6	7	8	9	10
1	***Phyllorhiza yurena* sp. nov. PV613528-31**	East China Sea	0									
2	*Phyllorhiza pacifica* KY610770	Thailand	0	-								
3	*Phyllorhiza pacifica* KY610774	Thailand	0	0	-							
4	*Phyllorhiza punctata* KY610771-3	Mexico	0.06%	0.06%	0.06%	0						
5	*Phyllorhiza punctata* HM194770, HG931673	Australia/Eastern Mediterranean	0.12%	0.11%	0.11%	0.17%	0					
6	*Mastigias papua* HM194796	Palau	0.30%	0.28%	0.28%	0.34%	0.40%	-				
7	*Versuriga anadyomene* HM194768	Palau	0.60%	0.57%	0.57%	0.63%	0.68%	0.57%	-			
8	*Cassiopea andromeda* KY610763	Mexico	0.96%	0.91%	0.92%	0.91%	1.02%	1.02%	0.85%	-		
9	*Cassiopea frondosa* KY610767	Panama	1.08%	1.02%	1.03%	1.03%	1.14%	1.14%	0.97%	0.23%	-	
10	*Lychnorhiza lucerna* KY610785	Nicaragua	2.18%	2.75%	2.75%	2.76%	2.87%	2.87%	2.69%	2.45%	2.57%	-

Sequences of the new species are annotated in bold.

## Data Availability

The data presented in this study are openly available in NCBI GenBank at https://www.ncbi.nlm.nih.gov/genbank/ (accessed on 12 May 2025).
